# Aberrant DNA methylation of genes regulating CD4+ T cell HIV‐1 reservoir in women with HIV

**DOI:** 10.1002/ctm2.70267

**Published:** 2025-03-11

**Authors:** Ke Xu, Xinyu Zhang, Kesava Asam, Bryan C. Quach, Grier P. Page, Deborah Konkle‐Parker, Claudia Martinez, Cecile D. Lahiri, Elizabeth F. Topper, Mardge H. Cohen, Seble G. Kassaye, Jack DeHovitz, Mark H. Kuniholm, Nancie M. Archin, Amir Valizadeh, Phyllis C. Tien, Vincent C. Marconi, Dana B. Hancock, Eric O. Johnson, Bradley E. Aouizerat

**Affiliations:** ^1^ Department of Psychiatry, School of Medicine Yale University New Haven Connecticut USA; ^2^ VA Connecticut Healthcare System West Haven Connecticut USA; ^3^ Department of Oral and Maxillofacial Surgery New York University New York New York USA; ^4^ Translational Research Center New York University New York New York USA; ^5^ GenOmics and Translational Research Center RTI International, Research Triangle Park North Carolina USA; ^6^ Fellow Program, RTI International, Research Triangle Park North Carolina USA; ^7^ Schools of Nursing, Medicine, and Population Health University of Mississippi Medical Center Jackson Mississippi USA; ^8^ Miller School of Medicine, Division of Cardiovascular Medicine University of Miami Miami Florida USA; ^9^ Department of Medicine, Division of Infectious Diseases Emory University School of Medicine Atlanta Georgia USA; ^10^ Department of Epidemiology, Bloomberg School of Public Health Johns Hopkins University Baltimore Maryland USA; ^11^ Department of Medicine, Stroger Hospital Cook County Health System Chicago Illinois USA; ^12^ Department of Medicine, Division of Infectious Diseases Georgetown University Washington District of Columbia USA; ^13^ Department of Medicine, Division of Infectious Diseases Downstate Health Sciences University Brooklyn New York USA; ^14^ Department of Epidemiology and Biostatistics University at Albany, State University of New York Rensselaer New York USA; ^15^ UNC HIV Cure Center University of North Carolina at Chapel Hill School of Medicine Chapel Hill North Carolina USA; ^16^ Department of Medicine, Division of Infectious Diseases University of North Carolina at Chapel Hill School of Medicine Chapel Hill North Carolina USA; ^17^ Department of Medicine University of California at San Francisco San Francisco California USA; ^18^ Hubert Department of Global Health, Rollins School of Public Health Atlanta Georgia USA; ^19^ Atlanta VA Medical Center Decatur Georgia USA

## Abstract

**Background:**

The HIV‐1 reservoir in CD4+ T cells (HR_CD4_) pose a major challenge to curing HIV, with many of its mechanisms still unclear. HIV‐1 DNA integration and immune responses may alter the host's epigenetic landscape, potentially silencing HIV‐1 replication.

**Methods:**

This study used bisulphite capture DNA methylation sequencing in CD4+ T cells from the blood of 427 virally suppressed women with HIV to identify differentially methylated sites and regions associated with HR_CD4_.

**Results:**

The average total HR_CD4_ size was 1409 copies per million cells, with most proviruses defective and only a small proportion intact. The study identified 245 differentially methylated CpG sites and 85 regions linked to HR_CD4_ size, with 52% of significant sites in intronic regions. Genes associated with HR_CD4_ were involved in viral replication, HIV‐1 latency and cell growth and apoptosis. HR_CD4_ size was inversely related to DNA methylation of interferon signalling genes and positively associated with methylation at known HIV‐1 integration sites. HR_CD4_‐associated genes were enriched on the pathways related to immune defence, transcription repression and host–virus interactions.

**Conclusions:**

These findings suggest that HIV‐1 reservoir is linked to aberrant DNA methylation in CD4+ T cells, offering new insights into epigenetic mechanisms of HIV‐1 latency and potential molecular targets for eradication strategies.

**Key points:**

- Study involved 427 women with HIV.
- Identified 245 aberrant DNA methylation sites and 85 methylation regions in CD4+ T cells linked to the HIV‐1 reservoir.
- Highlighted genes are involved in viral replication, immune defence, and host genome integration.
- Findings suggest potential molecular targets for eradication strategies.

## Graphical abstract

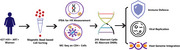

## 1 INTRODUCTION

The HIV‐1 reservoir (HR) has posed a major barrier to an HIV cure. The provirus persists in resting or memory CD4+ T cells^1^, ^2^ because of cellular biological conditions during the early stage of infection. These conditions include near‐silenced gene expression due to a lack of necessary host transcription factors, restrictive epigenetic features at the proviral promoter and sequestration of key transcription factors (like NFAT and NF‐κB).^3^, ^4^ Such conditions enable the HR to evade the immune system, posing challenges in curing HIV. Other cell types, such as monocytes, are also of interest for HR, but little is known about their role in HIV reservoir. Due to the presence of HR, cessation of anti‐retroviral therapy (ART) leads to HIV‐1 rebound usually within approximately 2 weeks.^5^ The HR size decays slowly with an estimated half‐life of 44 months for the total HR^6^ and 59 months for the intact provirus.^7^ At this decay rate, eradication would take over 60 years, necessitating lifelong ART for people with HIV (PWH).

In recent years, efforts to cure HIV have focused on latency reversal followed by immune‐mediated clearance of infected cells. However, latency reversal agents have failed to effectively reduce HR size, presenting a major challenge.^8^, ^9^ Another obstacle is the limited understanding of how HR establishes, maintains, and rebounds in CD4^+^ T cells before, during, and after ART discontinuation. Addressing these challenges is particularly important among women with HIV (WWH), whose immune response to HIV‐1 may differ from that of men. WWH exhibit lower HIV‐1 viral load during the early stage of HIV infection but show higher immune activation and faster progression compared with men with HIV,^10^, ^11^, ^12^ which may influence HR latency and size for WWH. Furthermore, sex hormones can modulate immune function. For example, oestrogen has been shown to enhance the activity of certain immune cells, including HIV‐1‐targeted CD4+ T cells.^13^, ^14^ Hormonal fluctuations such as those occurring during menopause can affect the dynamics of HIV infection and the establishment of viral reservoirs. The underlying mechanisms of HR among WWH have not been understood.

Emerging evidence shows that epigenetic regulation of both the viral and host genomes is crucial to establishing and maintaining HIV‐1 latency. Transcription of provirus depends upon the dynamic interaction between genomic alteration of the provirus and host immune activity.^15^ The HIV‐1 provirus genome reduces chromatin accessibility in latently infected cells.^16^ Mechanistically, two CpG sites in the promoter region of long terminal repeats (LTR) of the provirus are hypermethylated, mediated by the host methyl‐CpG binding domain protein 2 in infected CD4^+^ T cells.^17^ In contrast, some studies reported that methylation of LTR was not associated with HIV‐1 latency.^18^, ^19^ Research on the host genome's DNA methylation and HR is still in its infancy. Some studies have reported that hyper‐ and hypo‐methylated regions are associated with HR size and positively correlate with disease progression assessed by viral response to ART,^20^ but these findings are based on small sample sizes or ex vivo experiments, making it challenging to draw strong conclusions about in vivo host DNA methylation dynamics associated with HR.

Genome‐wide DNA methylation analysis with high‐density CpG profiling is a useful approach to comprehensively investigate the role of the host DNA methylome in HR size. Among the total reservoir in infected CD4^+^ T cells, only a small proportion contains intact provirus (∼1 per million cells) capable of full transcription activation but remain inactivated partially due to heterogenous integration landscape and epigenetic mechanisms.^21^, ^22^, ^23^ Most proviruses are defective because of apolipoprotein B mRNA editing enzyme catalytic polypeptide‐like 3G (*APOBEC3C*)‐mediated hypermutation,^24^ large internal deletions, packaging signal (Ψ) deletions, major splice donor mutations and inactivating point mutations.^25^ Most defective proviruses accumulate swiftly during early HIV‐1 infection.^24^ While previous studies have focused on intact reservoirs, recent research indicates that defective provirus can be activated to produce HIV‐1 transcripts when integrated at active transcription sites,^25^ and it can be recognised by cytotoxic T cells.^26^ Proviruses escape mutations and remain stable during ART,^27^ leading to chronic immune activation and inflammation, which are associated with the progression of HIV‐related comorbidities.^28^, ^29^, ^30^ Thus, it is plausible that host epigenome alterations occur in response to both intact and defective reservoirs after HIV‐1 integration. Characterisation of the perturbed host epigenome by intact and defective proviruses may help us discover new targets to eradicate the reservoir.

In this study, we examined the association of DNA methylation features with HR size in CD4^+^ T cells (HR_CD4_) from a cohort of WWH who were virally suppressed on ART (*N* = 427). DNA samples were extracted from isolated CD4^+^ T cells. Using a methylation capture sequencing (MC‐seq) method, we profiled DNA methylation sites across the epigenome and found 245 methylation sites associated with total HR_CD4_. The genes annotated for significant methylated sites were involved in HIV‐1 latency, inflammation and cell differentiation and harboured HIV‐1 integration sites. These findings provide new evidence on the potential roles of host epigenetic factors in HR size through type I interferon genes, immune activation and chromosome stability.

## 2 RESULTS

### 2.1 Participant characteristics and HIV‐1 latent reservoir detection

The study population was derived from the Women's Interagency HIV Study (WIHS) a multi‐centre interval cohort study of WWH and a comparison group of women without HIV infection in the United States.^31^, ^32^ In 2019, WIHS merged with the Multicenter AIDS Cohort Study (MACS) to form the MACS/WIHS Combined Cohort Study (MWCCS).^33^ Participants underwent follow‐up visits every 6 months, which included the collection of peripheral blood mononuclear cells (PBMC) and demographic, clinical, and ART treatment data. The 427 participants who met the inclusion and exclusion criteria were selected for the current study (see section *Methods*). Demographic and clinical characteristics are presented in Table 1.

**TABLE 1:** Demographic and clinical characteristics of participants.

Variable	Participants (*N* = 427)
Age (mean ± SD)	47.29 ± 8.22
Sex (F/M)	427/0
Ancestry (%)	
African American	76.3
European	12.4
Other	11
Smoking status (%)	
Current	79.9
Former	8.9
Never	11
Alcohol drinks per week (mean ± SD)	9.3 ± 26.5
On ART + undetected VL (%)	100
HR (mean ± SD)	
Total	1409 ± 1639
Intact	279 ± 463.4
5′‐depletion	600 ± 747.7
3′‐depletion	530 ± 885.7

Abbreviations: ART, anti‐retroviral therapy; HR, HIV‐reservoir; VL, viral load.

The average age was 47.3 ± 8.2 years; 76% of the participants self‐identified as African American non‐Hispanic, 12% as White non‐Hispanic and 11% as other races. Most participants (80%) currently smoked tobacco. All participants were on ART and had undetectable viral loads (below the assay lower limit of quantitation) for a minimum of 6 months, with an average duration of 2.1 years, up to the visit at which CD4+ T cells were isolated from PBMCs and were processed to estimate the HR_CD4_. The genomic DNA extracted from CD4+ T cells was subjected to bisulphite capture sequencing for DNA methylation profiling.

The HR_CD4_ quantification method for this cohort was detailed previously by Aouizerat et al.^34^ Briefly, HR_CD4_ was detected using droplet digital polymerase chain reaction (ddPCR). Intact proviral HIV DNA was measured using a modified intact proviral DNA assay (IPDA). The modified IPDA included the original primer sets targeting the Psi (*Y*) and *env* regions of the HIV‐1 provirus, and the addition of a primer set targeting a host gene (i.e., CD4+ T cells) to detect and adjust for DNA shearing, which results in underestimates of the HR size. The average HR_CD4_ size was 1409 ± 1639 copies per million CD4^+^ T cells. As expected, defective HIV‐1 proviruses were more common than intact HR_CD4_. The average intact HR_CD4_ was 279 ± 463 copies per million; defective HR_CD4_ was 531 ± 886 copies per million for APOBEC3G‐mediated mutations (3′‐defective HR_CD4_) and 600 ± 748 copies per million for large internal deletions (5′‐defective HR_CD4_).

### 2.2 Epigenome‐wide methylation characterisation of HR_CD4_

CD4^+^ T cells epigenome was profiled using bisulphite MC‐seq, yielding data on methylation of 3.2 million CpG sites. Quality control (QC) was conducted according to our previous report (see section *Methods*).^35^ Only CpG sites with a coverage greater than 10× depth were included. Approximately 2 million CpG sites were analysed after QC. Ancestry and chronological age were included in generalised linear regression models.

**FIGURE 1:**
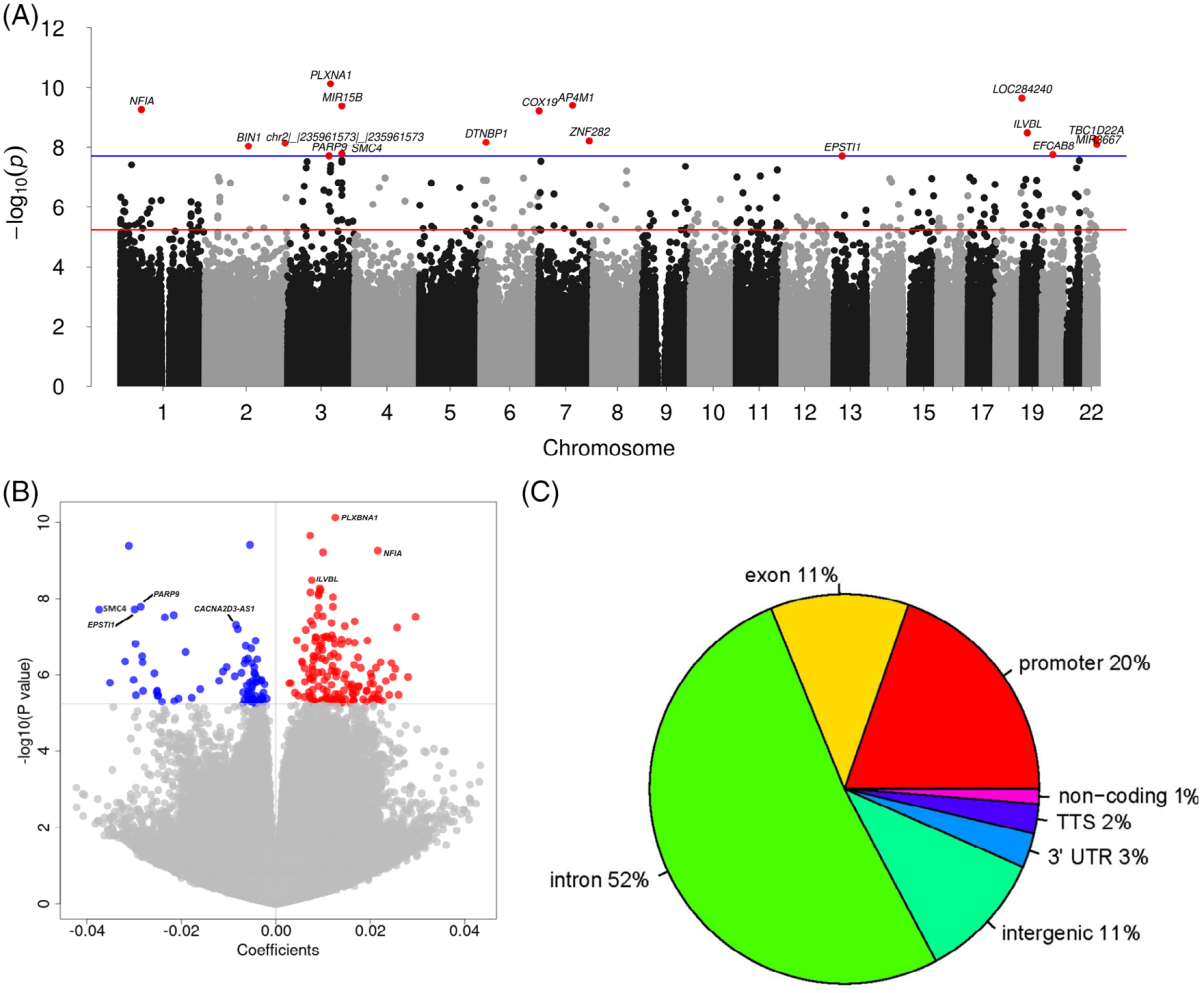
Epigenome‐wide association analysis of CD4^+^ T cell total HIV‐1 reservoir size: (A) Manhattan plot; red line indicates false discovery rate at 0.05 and blue line indicates Bonferroni *p* value at .05; (B) volcano plot showing positive and negative association of CpG sites with total HR; (C) proportion of CpG site annotations.

We found 245 differentially methylated sites associated with total HR_CD4_ size (all integrated virus DNA) across the epigenome (false discovery rate, FDR < 0.05) (Figure 1A and Table S1), suggesting that these regions are potentially involved in HIV‐1 establishment and/or persistence Among them, 67% (*n* = 165) were positively associated with HR_CD4_ size, meaning higher methylation is linked to greater HR_CD4_ size, while 33% (*n* = 80) were inversely associated with it (Figure 1B). A substantial proportion of these methylation sites (89%) were annotated within or near genes, with the rest (11%) in intergenic regions. Within gene regions, 52% of CpG sites were in introns, 20% in promoter regions and 11% in exons (Figure 1C). Additionally, 26% of the associated sites mapped to CpG islands, with the rest in non‐island regions (open sea 34%, shore 30% and shelf 9%). Notably, although most of the observed associations were positive, 50% of the methylation sites in the promoter regions were negatively associated with total HR_CD4_.

We further examined whether the total HR_CD4_‐associated CpG sites were also significantly associated with intact and defective HR_CD4_. Among the 245 significant CpG sites for total HR_CD4_, 38 were nominally associated with intact or defective HR_CD4_ (*p* < .05). For example, *SMC4* on chromosome 3 at position 160119770 was negatively associated with total (*t* = −5.79, *p* = 1.64e−08), intact (*t* = −4.0, *p* = 7.63e−05) and 5′‐defective (*t* = −4.78, *p* = 2.64e−06) HR_CD4_. *PARP9* on chromosome 3 at position 122281940 was also negatively associated with total (*t* = −5.76, *p* = 1.92e−08), intact (*t* = −3.19, *p* = 1.57e−03), 5′‐defective (*t* = −3.66, *p* = 2.93e−04) and 3′‐defective (*t* = −4.28, *p* = 2.39e−05) HR_CD4_. *ISG15* on chromosome 1 at position 949432 was negatively associated with total (*t* = −5.14, *p* = 4.66e−07), intact (*t* = −3.39, *p* = 7.90e−04) and 5′‐defective (*t* = −4.53, *p* = 8.24e−06) HR_CD4_. These genes were previously reported to be involved in interaction with HIV‐1 tau protein (*SMC4*),^36^ HIV‐1 induced apoptosis (*PARP*)^37^ and HIV‐1 replication (*ISG15*),^38^ which may contribute to HIV‐1 latency.

Notably, considering the potential impact of menopause on DNA methylation and size, we conducted a secondary analysis to examine differences in HR size and methylation levels across 245 CpG sites between pre‐ and post‐menopausal women, as well as interactions between methylation of 245 sites and HR size by menopausal status. We found no significant differences in HR size between pre‐ and post‐menopausal women. However, 12 out of 245 CpG sites showed nominally significant differences (*p* < .05) between pre‐ and post‐menopausal women. More interestingly, 14 of 245 CpG sites exhibited interactive effects between menopause status and HR size (Table S2). For example, methylation of *ELAVL2* was negatively correlated with HR among pre‐menopausal women but positively correlated among post‐menopausal women (*p* = .002) (Figure S1, left). Although there is no direct evidence linking *ELAVL2* to menopause, this gene encodes an RNA‐binding protein highly expressed in ovaries, testes and neurons. Genetic variants in *ELAVL2* have been linked to primary ovarian insufficiency.^39^ Additionally, methylation of a CpG site on the promoter of *LMAN2 (Lectin, Mannose Binding 2)* was negatively correlated with HR among pre‐menopausal women but positively correlated among post‐menopausal women (*p* = 6.8e−04; Figure S1, right), with a nominally significant association with menopause status (*p* = .03). *LMAN2* encodes a type I transmembrane lectin associated with poor prognosis in breast cancer.^40^

Similarly, to investigate whether cigarette smoking influences the association between HR and DNA methylation, we examined the interactive effects between HR and current smoking status. We identified 14 nominally significant CpG sites affected by the interaction between smoking and HR size (*p* < .05) (Table S3). For example, methylation of a CpG site on *TNFRS13C* was increased among smokers with higher HR size (Figure S2).

### 2.3 Differentially methylated regions were significantly associated with total HR_CD4_

To find significant differentially methylated regions (DMR), we conducted an analysis using the ‘bump hunting’ approach (see section *Methods*). This analysis found DMRs containing at least 2 CpG sites associated with total HR_CD4_. We found 85 significant DMRs across 22 chromosomes (Table 2). Among them, 28 DMRs were negatively associated with HR_CD4_ while 57 DMRs were positively associated.

**TABLE 2:** Differentially methylated regions associated with total HIV‐1 reservoir in CD4^+^ T cells.

Chr	Start	End	# CpGs	*Gene*	*t*	*p*	FDR
1	61545077	61545123	2	*NFIA*	5.4	1.1e−09	9.2e−08
3	160119633	160122505	12	*MIR16‐2, MIR15B, SMC4*	−5.0	4.7e−09	2.0e−07
3	122281883	122281941	2	*DTX3L, PARP9*	−5.4	3.8e−08	1.1e−06
13	43565289	43565401	3	*EPSTI1*	−4.7	5.8e−08	1.2e−06
14	74250898	74250927	2	*ELMSAN1*	4.8	2.9e−07	4.1e−06
3	54888035	54888057	6	*CACNA2D3*	−4.4	2.9e−07	4.1e−06
2	43398273	43398277	2	–	5.1	5.4e−07	6.5e−06
7	44924186	44924194	2	*PURB*	−4.5	6.7e−07	7.1e−06
1	12676155	12676183	2	*DHRS3*	5.0	9.4e−07	7.7e−06
19	41815093	41815101	3	*CCDC97*	4.5	1.0e−06	7.7e−06
10	88852547	88852560	2	*GLUD1*	4.6	1.1e−06	7.7e−06
1	119522623	119522631	2	*TBX15*	4.5	1.1e−06	7.7e−06
1	949394	949433	3	*ISG15*	−4.6	1.2e−06	7.7e−06
11	118790174	118790223	3	–	4.6	1.4e−06	7.7e−06
1	207996299	207996461	3	–	4.8	1.4e−06	7.7e−06
17	7381527	7381577	2	*ZBTB4*	4.5	2.4e−06	1.3e−05
21	42798945	42799143	7	*MX1*	−4.4	2.7e−06	1.3e−05
15	44829519	44829525	2	*EIF3J, EIF3J‐AS1*	−4.5	3.0e−06	1.4e−05
1	207996636	207996676	2	–	4.7	3.3e−06	1.4e−05
9	134139856	134139880	3	*FAM78A*	4.2	3.3e−06	1.4e−05
10	22612799	22613027	2	*COMMD3‐BMI1, BMI1*	4.5	3.4e−06	1.4e−05
22	18635396	18635462	3	*USP18*	−4.4	3.8e−06	1.4e−05
16	4714768	4714796	3	*MGRN1*	4.5	4.1e−06	1.4e−05
2	37551058	37551083	5	–	4.6	4.1e−06	1.4e−05
11	75062095	75062115	2	*ARRB1*	4.4	6.3e−06	2.1e−05
21	42797710	42797717	2	*MX1*	−4.5	7.6e−06	2.4e−05
14	50159543	50159550	2	*KLHDC1*	4.4	8.7e−06	2.6e−05
11	121324233	121324511	3	*SORL1*	4.3	8.7e−06	2.6e−05
19	54876395	54876448	2	*LAIR1*	4.2	9.3e−06	2.6e−05
1	12538489	12538543	4	*VPS13D*	4.4	9.3e−06	2.6e−05
1	949698	949878	12	*ISG15*	−4.2	1.1e−05	2.8e−05
1	949563	949599	4	*ISG15*	−4.3	1.1e−05	2.8e−05
15	69707926	69707938	2	*KIF23*	−4.4	1.2e−05	3.1e−05
17	47437978	47438153	3	*ZNF652, LOC102724596*	4.2	1.4e−05	3.4e−05
15	55562701	55562788	4	*RAB27A*	−4.1	1.7e−05	4.0e−05
22	27068060	27068092	4	*MIAT, MIATNB*	4.2	1.8e−05	4.2e−05
4	38666920	38667432	2	*KLF3‐AS1, KLF3*	4.3	1.8e−05	4.2e−05
12	120700363	120700438	3	*PXN*	4.0	2.4e−05	5.2e−05
9	6716463	6716482	4	–	−4.2	2.5e−05	5.2e−05
17	79265630	79265645	2	*SLC38A10*	4.1	2.5e−05	5.2e−05
4	103682822	103682824	2	*MANBA*	4.2	2.5e−05	5.2e−05
21	45509115	45509120	2	*TRAPPC10*	4.3	2.7e−05	5.3e−05
11	614844	614857	3	*IRF7*	−4.1	2.8e−05	5.4e−05
1	949479	949508	3	*ISG15*	−4.2	2.8e−05	5.4e−05
10	90641813	90642005	3	*STAMBPL1*	−4.2	2.9e−05	5.5e−05
14	105751631	105751638	2	*BRF1*	4.1	3.1e−05	5.6e−05
22	39637150	39637172	4	*PDGFB*	4.1	3.2e−05	5.6e−05
1	226069169	226069191	2	*TMEM63A*	4.1	3.2e−05	5.6e−05
19	4380572	4380611	2	*SH3GL1*	4.1	3.3e−05	5.6e−05
3	43385089	43385113	2	*SNRK*	4.2	3.4e−05	5.6e−05
14	24631374	24631383	2	*IRF9*	−4.2	3.4e−05	5.6e−05
6	137630457	137630478	2	–	4.2	3.4e−05	5.6e−05
11	614567	614763	2	*IRF7*	−4.2	3.7e−05	5.9e−05
9	6716396	6716425	5	–	−4.1	3.9e−05	6.0e−05
17	42462262	42462268	2	*ITGA2B*	4.1	4.4e−05	6.7e−05
20	62530409	62530421	2	*DNAJC5*	4.1	4.7e−05	6.8e−05
2	43398215	43398245	2	–	4.1	4.7e−05	6.8e−05
2	173294414	173294494	2	*ITGA6*	4.0	4.8e−05	6.8e−05
21	45509391	45509401	3	*TRAPPC10*	4.1	4.8e−05	6.8e−05
22	38610341	38610350	2	*MAFF*	4.1	4.9e−05	6.8e−05
9	6716209	6716228	2	–	−4.1	4.9e−05	6.8e−05
11	61213606	61213621	2	*SDHAF2*	4.1	5.2e−05	7.0e−05
11	74975246	74975248	2	*ARRB1*	4.0	5.3e−05	7.1e−05
17	7381831	7381857	2	*ZBTB4*	4.1	5.6e−05	7.3e−05
2	71681539	71681542	2	*DYSF*	4.0	6.1e−05	7.9e−05
19	19281257	19281272	2	*MEF2BNB‐MEF2B, MEF2B*	4.0	6.3e−05	8.0e−05
11	109962729	109962762	2	*ZC3H12C*	4.0	6.8e−05	8.5e−05
21	45509454	45509457	2	*TRAPPC10*	4.0	6.8e−05	8.5e−05
14	61109826	61109857	2	–	4.0	7.1e−05	8.7e−05
4	85416937	85416953	2	*NKX6‐1*	3.9	7.9e−05	9.5e−05
16	53535169	53535242	2	*AKTIP*	3.9	9.0e−05	1.1e−04
1	154318280	154318316	2	*ATP8B2*	3.9	9.7e−05	1.1e−04
8	133098472	133098504	2	*HHLA1*	−3.9	1.1e−04	1.2e−04
17	37308830	37308847	2	*PLXDC1*	3.9	1.1e−04	1.3e−04
9	6716240	6716271	2	–	−3.9	1.2e−04	1.3e−04
6	168714672	168714675	2	*DACT2*	3.9	1.3e−04	1.4e−04
9	6716324	6716351	3	–	−3.9	1.3e−04	1.4e−04
9	79791171	79791179	2	*VPS13A*	3.9	1.4e−04	1.5e−04
3	122401131	122401302	3	*PARP14*	−3.8	1.4e−04	1.5e−04
5	142435222	142435443	2	*ARHGAP26*	−3.8	1.5e−04	1.5e−04
15	85177989	85178063	2	*SCAND2P*	3.8	1.5e−04	1.5e−04
1	79090602	79092806	2	*IFI44L*	−3.8	1.5e−04	1.5e−04
21	38065618	38065639	2	–	3.8	1.9e−04	1.9e−04
5	17219328	17219331	2	*BASP1*	3.8	1.9e−04	1.9e−04

**FIGURE 2:**
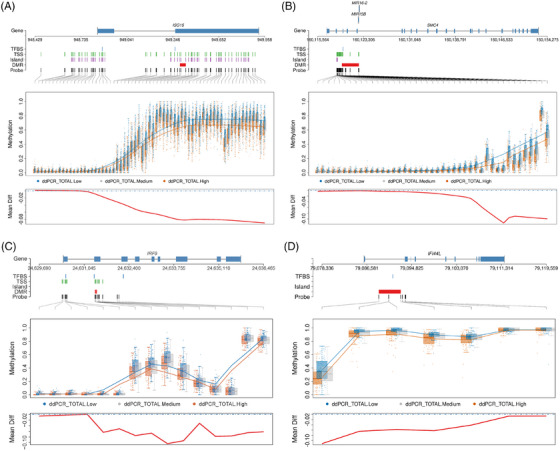
Differential methylation regions on four genes associated with HIV‐1 reservoir size. (A) Interferon‐stimulated gene 15 (*ISG15*); (B) structural maintenance of chromosomes 4 (*SMC4*); (C) interferon regulatory factor 9 (*IRF9*); (D) interferon‐induced protein 44 like (*IFI44L*). ddPCR‐total‐low: bottom 25th percentile HIV‐1 reservoir size; ddPCR‐total‐medium: medium HIV‐1 reservoir size; ddPCR‐total‐high: top 25th percentile HIV‐1 reservoir size.

Notably, four DMRs on *ISG15*, spanning 21 CpG sites, were significant, including one region harbouring 12 CpG sites. All four regions on *ISG15* were inversely associated with HR_CD4_ (Figure 2A). These CpG sites were either located in the TSS region or exons, suggesting that the aberrant methylation of *ISG15* may regulate *ISG15* expression, which is associated with HR. Another large significant DMR was located on chromosome 3 at *SMC4*, spanning 2872 base pairs and harbouring 12 CpG sites (Figure 2B). Similarly, this DMR was also inversely associated with HR_CD4_. Other negatively HR_CD4_‐associated DMR were in genes such as *PARP9, MX1, EPSTI1, USP18, IRF9, IFI44L* and *IRF7* (Figure 2C,D). Together, consistent with the findings in EWAS for individual CpG association, the results from DMR analysis further demonstrate that dysregulation of these genes (e.g. *ISG15, SMC4, PARP9, MX1, EPSTI1, USP18, IRF9, IFI44L* and *IRF7*) through methylation may indicate that HIV‐1 avoids immune detection and eradication in CD4+ T cells, allowing it to maintain its latent status.

### 2.4 Total HIV‐1 CD4+ T cell reservoir‐associated DNA methylation is related to HIV‐1 integration

Because half of the HR_CD4_‐associated differentially methylated sites were located in intronic regions, we explored whether our 245 methylation sites were enriched in known HIV‐1 genic integration sites by compiling HIV‐1 integration sites reported across three recent studies.^41^, ^42^, ^43^ We found that a subset of the genes harbouring significant CpG sites were targets of HIV‐1 integration. Methylation at most of these integration sites was positively associated with HR_CD4_ size.

**FIGURE 3:**
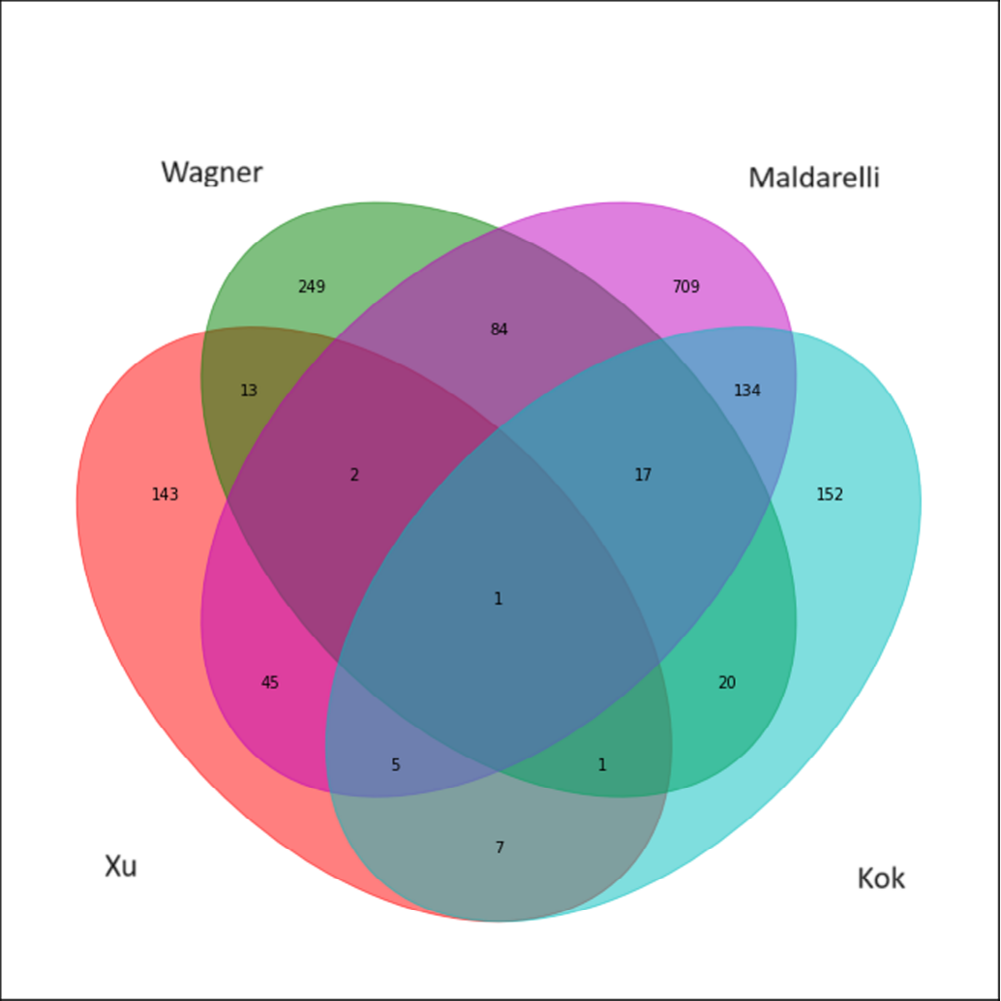
The number of significant genes associated with the HIV‐1 reservoir found in this study (Xu) and its overlap with the previously identified HIV‐1 integration sites in three other studies by Wagner et al., Maldarelli et al. and Kok et al.

One gene, *IMPDH2*, was a common HIV‐1 integration site between our study and the three previous studies.^41^, ^42^, ^43^ A CpG site on chromosome 3 at position 49066890, which was in the promoter region of *IMPDH2*, was positively associated with HR_CD4_ size. We also found that 23 HR_CD4_‐associated genes overlapped with HIV‐1 integration sites reported by Kok et al.^41^ Also, a few other genes were shared between our study and the other two previous studies,^41^, ^42^ including *NFIA, TMTC3, SPPL3, DLEU2, ELMSAN1* and *ACSF3*. A total of 53 genes overlapped with HIV‐1 integration sites reported by Maldarelli et al.^43^ (e.g., *ISG15, TTC19, KCNG1, EIF3J, COX7A2L, TTC19, LAIR1, PARP9, JAK1* and *CCDC97*) and 17 with sites reported by Wagner et al.^42^ (e.g., *TTBK2, CDK16, ATP5PO* and *TFDP1*) (Figure 3).

**FIGURE 4:**
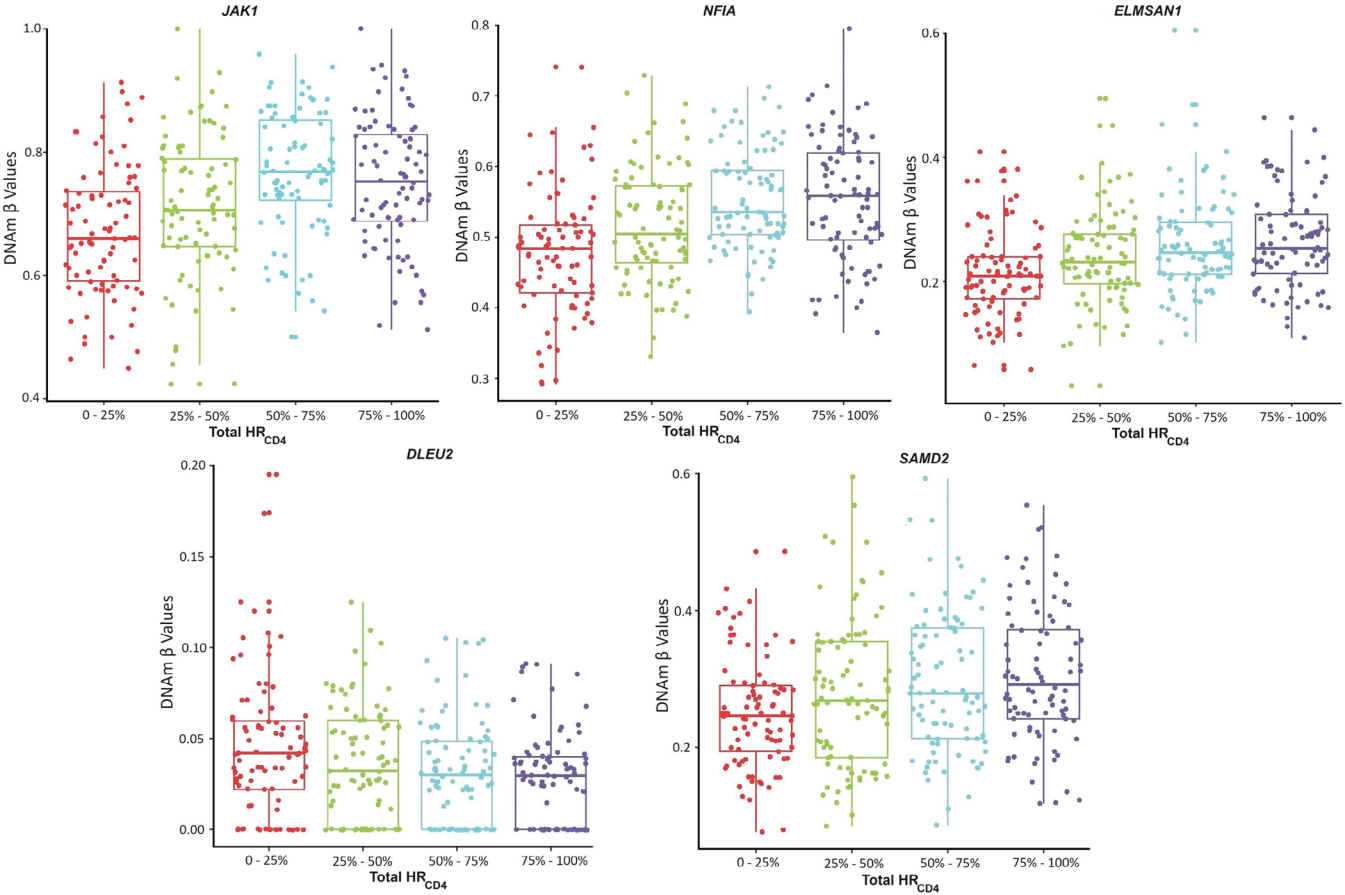
CpG sites that were differentially methylated between the high and low HIV‐1 reservoir sizes on genes involved in HIV‐1 integration sites. (A) *JAK1* (chr1:6550357 *p* = 5.74e−07); (B) two CpG sites on *NFIA* (chr1:6145122, *p* = 5.49e−10 and chr1:61542330, *p* = .00004); (C) three CpG sites on *ELMSAN1* (chr14:74244367, *p* = 6.62e−05; chr14:74250897, *p* = 1.43e−07; chr14:74250926, *p* = .00004) and (D) two CpG sites on *DLEU2* (chr13:50653658: *p* = .00001 and chr13:506554306: *p* = 1.88e−06); (E) SMAD2 (chr18:45458622: *p* = .0000128).

The most frequently reported HIV‐1 integration site, *BACH2*,^42^, ^43^ was nominally associated with HR_CD4_ (*p* = .001) in our study. Additionally, several other previously reported integration sites displayed consistent patterns of differential methylation, with higher methylation levels in the top 25th percentile of total HR_CD4_ size compared with the lowest 25th percentile. For example, a CpG site at *JAK1* had 15% higher methylation in the top 25th percentile for HR_CD4_ size, compared with the lowest 25th percentile (*chr1 65503557, p* = 5.74e−07) (Figure 4A). Similarly, two CpG sites at *NFIA* showed 7 and 10% higher methylation levels in the top 25th percentile (*chr1 61542330, p* = .00004; *chr1 61545122*, *p* = 5.49e−10) (Figure 4B). Other examples include three CpG sites for *ELMSAN1* (*chr14 74244367*, *p* = 6.62e−05; *chr14 74250897*, *p* = 1.43e−07; *chr14 74250926*, *p* = .00004) (Figure 4C), two CpG sites for *DLEU2* (*chr13 50653658*, *p* = .00001; *chr13 506554306*, *p* = 1.88e−06) (Figure 4D) and one CpG site for *SMAD2* (*chr18 45458622*, *p* = 1.28e−05) (Figure 4E). The results indicate that aberrant DNAm on the genes involved in chromatin remodelling and maintaining genomic stability.

### 2.5 Total HR_CD4_‐associated CpG sites are enriched in pathways relevant to HIV‐1 latency

**FIGURE 5:**
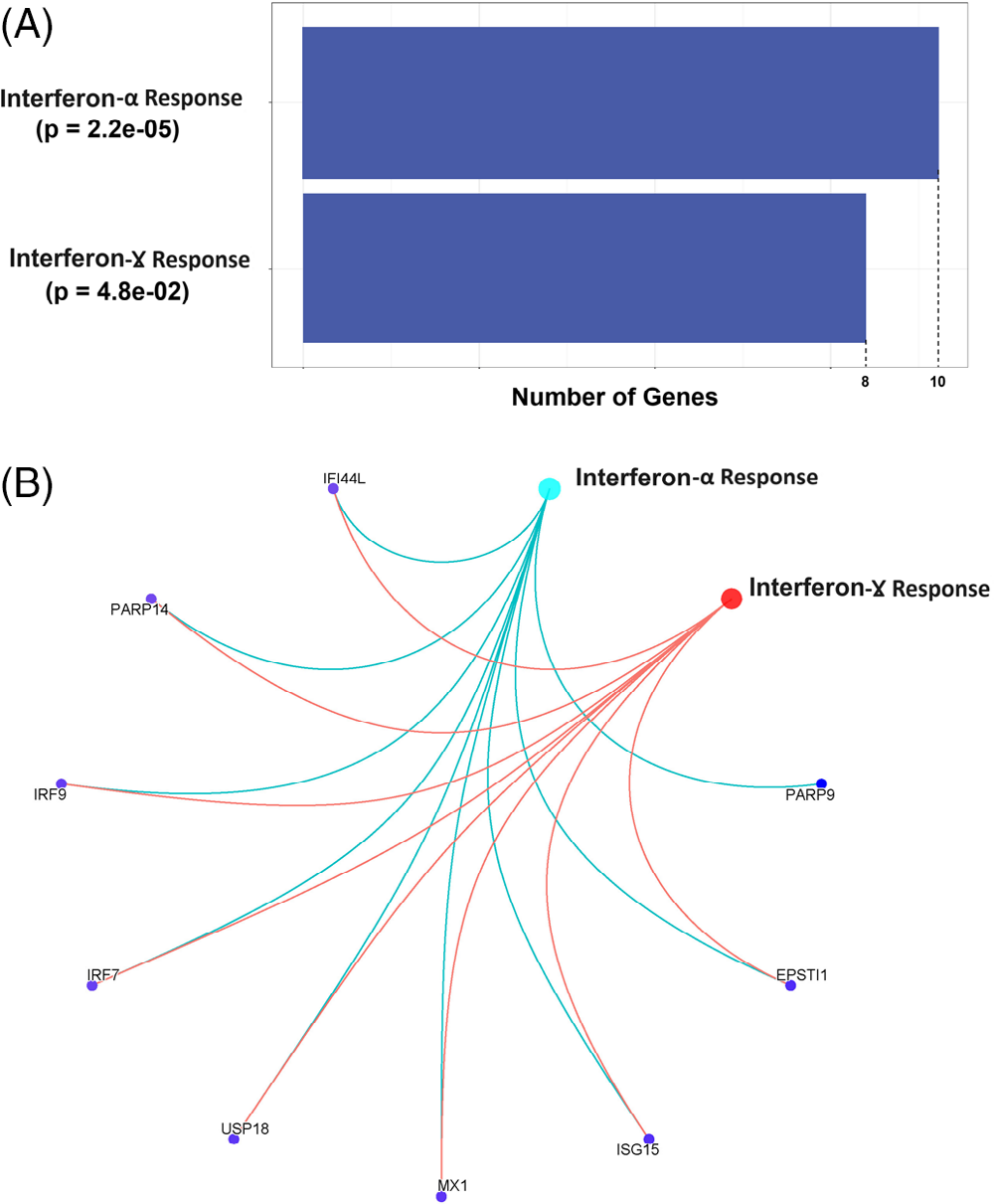
Significant pathways enriched on Hallmark gene set. (A) IFN‐α and IFN‐γ pathways; (B) HIV‐1 reservoir‐associated genes on those pathways.

We performed gene enrichment analysis for the set of genes close to or harbouring 245 aberrant methylation sites using the human MSigDB database (https://www.gsea‐msigdb.org/gsea/msigdb/), which includes 33591 genes from nine major collections. In the hallmark gene set, we found significant enrichment for HR_CD4_‐associated genes in the interferon α response (gene ratio: 16/79; *p*_adj _= 1.50e−10) and interferon γ response (gene ratio: 16/79; *p*_adj _= 3.45e−06) (Figure 5A,B and Table 3). In the interferon α gene set, nine genes were associated with HR_CD4_, with eight of these overlapping between the ‘interferon α response’ and ‘interferon γ response’ gene sets. These shared genes were *USP18, MX1, ISG15, IRF7, IFIT3, IFI44L, IFI27* and *EPST1*, all of which reached epigenome‐wide significant association with total HR_CD4_. Four of these genes are involved in the regulation of inflammatory function (i.e., *IRF7, IFIT3, IFI44L* and *IFI27*).

**TABLE 3:** Significantly perturbed pathways associated with CD4+ T‐cell total HIV‐1 reservoir size.

Pathway	Gene ratio	*p*	*q*
**Hallmark pathways**			
Interferon‐α response	10/97	2.2e−05	1.0e−03
Interferon‐γ response	8/200	4.8e−02	7.7e−01
**GO molecular function pathways**			
Tau protein binding	5/272	1.1e−03	3.0e−01
Vinculin binding	3/272	1.2e−03	3.0e−01
DNA binding transcription repressor activity	13/272	6.1e−03	6.7e−01
DNA binding transcription activator activity	16/272	1.1e−02	6.7e−01
DNA binding transcription factor binding	16/272	1.2e−02	6.7e−01
Aromatic amino acid transmembrane transporter activity	2/272	1.7e−02	6.7e−01
Oligopeptide transmembrane transporter activity	2/272	1.7e−02	6.7e−01
Intermediate filament binding	2/272	2.0e−02	6.7e−01
Peptide transmembrane transporter activity	2/272	2.0e−02	6.7e−01
Amino acid transmembrane transporter activity	5/272	2.0e−02	6.7e−01
Transcription factor binding	18/272	2.2e−02	6.7e−01
Cell adhesion molecule binding	17/272	2.2e−02	6.7e−01
Organic anion transmembrane transporter activity	8/272	2.3e−02	6.7e−01
2‐Oxoglutarate‐dependent dioxygenase activity	4/272	2.3e−02	6.7e−01
Glycosyltransferase activity	10/272	2.3e−02	6.7e−01
Β‐1‐3‐Galactosyltransferase activity	2/272	2.6e−02	6.7e−01
Proton transmembrane transporter activity	6/272	2.7e−02	6.7e−01
Glycosaminoglycan binding	9/272	2.9e−02	6.7e−01
Mechanosensitive ion channel activity	2/272	3.0e−02	6.7e−01
l‐Amino acid transmembrane transporter activity	4/272	3.2e−02	6.7e−01
Demethylase Activity	3/272	3.2e−02	6.7e−01
Organic Acid Transmembrane Transporter Activity	7/272	3.4e−02	6.7e−01
Cadherin binding	11/272	3.4e−02	6.7e−01
Insulin‐like growth factor receptor binding	2/272	3.4e−02	6.7e−01
Heparin binding	7/272	3.6e−02	6.7e−01
Core promoter sequence‐specific DNA binding	3/272	3.7e−02	6.7e−01
G‐protein activity	3/272	3.7e−02	6.7e−01
Monosaccharide binding	4/272	3.8e−02	6.7e−01
Magnesium ion transmembrane transporter activity	2/272	3.8e−02	6.7e−01
Protein self‐association	4/272	4.2e−02	6.7e−01
NAD+ binding	2/272	4.2e−02	6.7e−01
Opsonin binding	2/272	4.2e−02	6.7e−01
Mannose binding	2/272	4.7e−02	6.9e−01
Protein serine threonine tyrosine kinase activity	3/272	4.9e−02	6.9e−01

**FIGURE 6:**
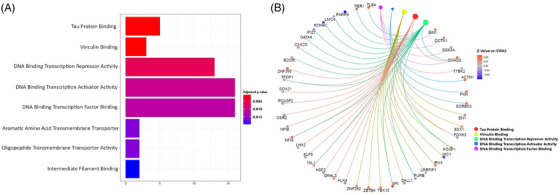
Gene set enrichment analysis results using GO molecular function terms. (A) significant pathways; (B) HIV‐1 reservoir‐associated genes enriched on eight significant pathways; (C) the relationship between the top five pathways.

Gene set enrichment analysis employing Gene Ontology (GO) molecular function terms resulted in 34 HR_CD4_‐associated gene sets that were nominally significant but did not remain significant after multiple‐testing correction. Nevertheless, these gene sets were highly relevant to HIV‐1 pathogenesis (Figure 6A,B and Table 3). The top‐ranked gene sets included ‘tau protein binding’ (*p* = .001), ‘vinculin binding’ (*p* = .001), ‘transcription factor binding’, ‘DNA binding transcription repressor activity’ (*p* = .006), ‘DNA binding transcription factor binding’ (*p* = .01) and ‘interferon binding’ (*p* = .02). Nine genes were enriched in more than one gene set (i.e., *SMAD2, FOXK2, FOXP1, IRX3, PURB, SK1, ISL1, NFIA* and *TFDP1*).

Interestingly, *SMAD2* connects the ‘tau‐protein binding’ pathway to the ‘DNA binding transcription factor’ and ‘DNA binding transcription activator activity’ pathways. A methylation site at *SMAD2* was positively associated with HR_CD4_ (*t* = 4.43, *p* = 1.28e−05). *SMAD2* is responsible for transmitting the signal of transforming growth factor (TGF)‐beta,^44^ which plays a crucial role in regulating various cellular processes like cell proliferation, differentiation and apoptosis.

## 3 DISCUSSION

Here, we report the first epigenome‐wide association study on total HR_CD4_, highlighting significant new insights into DNA methylation mechanisms involved in the HR. Using bisulphite capture sequencing on samples collected from 427 women with virally suppressed HIV‐1 infection, we comprehensively characterised the epigenomic landscape of HR_CD4_ over 2 million CpG sites. We found 245 DNA methylation sites and 87 regions in CD4+ T cells associated with total HR_CD4_ size. Notably, genes and pathways related to significant CpG sites were involved in the host immune responses, particularly type I interferon genes and JAK1, which regulate HIV‐1 replication and integration. We also observed HR_CD4_‐associated aberrant methylation on the genes involved in chromosome stability, which may contribute to HIV‐1 integration. Our results, derived from the largest sample size reported to date, reveal specific methylation mechanisms for HIV‐1 persistence that may inform the potential target eradication strategies for HIV care.

Consistent with previous findings,^25^ we found that the majority of the integrated DNA proviruses were defective. Recent studies have reported that both intact and defective integrated HIV‐1 DNA proviruses remain transcriptionally active under suppressive ART, contributing to prolonged immune activation^45^, ^46^, ^47^ and greater comorbidity among PWH. Epigenetic regulation of host factors plays a critical role in HIV‐1 transcriptome activation and silencing. In line with our current understanding of the mechanisms of HIV‐1 pathogenesis, we found epigenetic alterations in genes involved in immune defence, interactions with viral proteins and HIV‐1 integration, suggesting that aberrant DNA methylation of these genes is linked to HIV‐1 persistence.

Notably, we found aberrant DNA methylation of multiple type I interferon genes associated with total HR_CD4_ size. For example, four significant CpG sites for the interferon‐induced protein *ISG15* were inversely associated with HR_CD4_. A 445 base pairs region in *ISG15* containing multiple CpG sites was significantly less methylated in the highest total HR_CD4_ size quartile compared with the lowest quartile. Similarly, three regions in *interferon regulatory transcription factor 7 (IRF7)* encompassed 20 CpG sites that were less methylated in the highest compared with the lowest HR_CD4_ quartile. Additionally, a region spanning 4 CpG sites on *IRF9* was significantly less methylated in the highest compared with the lowest HR_CD4_ quartile. Other interferon‐induced genes such as *IFI44L, MX1*, *PARP9* and *USP18* consistently showed an inverse relationship between CpG methylation and HR_CD4_ size. Interferon genes are known to function as antiviral signalling molecules, regulate immune function and induce apoptosis. As a result, it is presumed that increased expression of interferon genes due to hypomethylation may enhance restriction factor activity, resulting in limited HIV‐1 replication and maintaining HR_CD4_ in a latent state. Furthermore, interferons also promote increased CD4+ T cell localisation and HIV coreceptor expression, potentially enlarging the reservoir through seeding other cells. Our results support this complex and critical role of interferons in HIV‐1 replication and latency.^48^

Another notable finding is that a hypermethylated CpG site at *JAK1* was associated with larger HR_CD4_ size. *JAK1* is a critical mediator of immune activation and is involved in HIV‐1 latency. Previous studies have shown that *JAK1* interacts with the HIV‐1 transcriptome and is essential for its life cycle. HIV‐1 envelop surface glycoprotein gp120^49^, ^50^ has been found to induce the expression of *JAK1* through the Tat‐mediated activity of PIK3R1.^51^ Additionally, other studies have found that reduced expression levels of *JAK1* are associated with the inhibition of HIV LTR‐beta‐gal activity.^52^ A clinical trial showed that ruxolitinib, a JAK1/2 inhibitor, significantly decreased immune activation markers in PWH and reduced HRs.^53^ Thus, the aberrant methylation of *JAK1* in relation to HR_CD4_ size in this study further supports the significant role of *JAK1* in HIV‐1 latency.

HIV‐1 genome tends to integrate into highly expressed genes and genes involved in clonal expansion and cell cycle control.^41^ In this study, several genes involved in HIV‐1 integration and latency showed significant association with HR_CD4_. For example, the methylation of *NFIA*, a transcription factor highly expressed in HIV‐1 latently infected CD4+ T cells,^54^ was positively associated with HR_CD4_ size, with the methylation being more pronounced in the highest HR_CD4_ size quartile.

Aberrant DNA methylation associated with HR_CD4_ was also discovered in genes responsible for chromosome stability, such as *SMC4*. *SMC4* is part of a family of genes that encode the structural maintenance of chromosome (SMC) proteins, which are parts of the cohesion and condensin complexes essential for chromatid pairing and chromosome segregation during mitosis.^55^ Dysfunction of SMC proteins may increase vulnerability to HIV‐1 integration into the host genome. They also play critical roles in regulating transcription and ATPase domains and are crucial for maintaining chromosome structure and the repair and stability of DNA. *SMC4* is also a positive regulator of innate inflammatory immune responses and cell proliferation,^56^ which are key host factors for HIV‐1 persistence. It is also believed that in T cells, *SMC4* interacts with HIV‐1 Tat protein, which controls HIV‐1 DNA replication and nuclear architecture.^36^ The observed aberrant DNA methylation associated with HR_CD4_ size in *SMC4*, further supports its involvement in the establishment and maintenance of HR.

Of note, it is well documented that cigarette smoking has remarkable impacts on DNA methylome.^57^ We previously reported that cigarette smoking was strongly associated with DNA methylation among PWH.^58^ In this study, we found significant differential effects of DNA methylation on HR size between current smokers and non‐smokers, suggesting that smoking may influence the relationship between DNA methylation and HR size. The causal relationship among smoking, DNA methylation and HR size warrants further investigation in a larger sample population.

We acknowledge some limitations in our study. First, we were unable to find epigenome‐wide significant methylation sites specific for intact or defective reservoirs, necessitating more sensitive detection methods. Additionally, sample input requirements and technical challenges of studying HR_CD4_ in a large sample of PWH precluded the measurement of the functional HR_CD4_ (e.g., QVOA) in conjunction with IPDA. A precise measurement of HIV‐1 latency requires quantifying cellular HIV‐1 RNA transcription. Measuring possible HR in a subtype of CD4+ T cells in a large sample is challenging. As a result, confirming the identified methylation sites for HR_CD4_ using single‐cell methylation profiles is needed. In addition, we are unable to rule out the potential confounding effect of cigarette smoking on DNA methylation marks, as most of the participants were smokers. Our cross‐sectional study design limits the ability to assess the causal relationship between altered DNA methylation and HR size. Both longitudinal and ex vivo studies are needed to figure out the causality between DNA methylation and reservoir size. Last, this study did not include men. Future research is also needed to replicate these findings in men living with HIV and explore the dynamic interaction between the genes found in this study and the HIV‐1 genome in reservoir establishment and maintenance as well as sex difference.

In summary, this comprehensive characterisation of the epigenome concerning HR_CD4_ sheds light on the epigenetic mechanisms underlying HIV‐1 persistence. Most importantly, these findings offer new gene targets such as *ISG15*, *PARP*, *NFIA* and *SMC4* for an in‐depth exploration of host responses to the HR. These targets could potentially serve as molecular points of interest for the development of novel treatments.

## 4 METHODS

### 4.1 Ethics approval and consent to participate

The study was approved by the Committee of Human Research Subject Protection at Yale University and the Institutional Research Board Committee of the Connecticut Veteran Healthcare System. Informed consent was provided by all MWCCS participants via protocols approved by institutional review committees at each affiliated institution.

### 4.2 Participants and HR detection

Clinical data and specimens used in this study were collected by MWCCS.^33^ MWCCS is the largest observational cohort of HIV infection in the United States. The inclusion criteria were WWH who had undetectable HIV‐1 viral load (below the limit of quantitation for the assay) on ART treatment for at least 6 months.^59^ MWCCS is a longitudinal cohort study and the cohorts span the changes in the lower limit of detection for the assay, ranging from 400 (the earliest assay) to 20 (the current assay). The different threshold was adjusted in the analytical model below. A total of 427 WWH who met the criteria were selected for the study. The average duration of ART treatment was ∼2.1 years among the participants. CD4^+^ T cells were isolated by negative bead selection using the DynaBeads Untouched Human CD4 kit. Genomic DNA was then extracted from CD4^+^ T cells using magnetic bead‐based nucleic acid isolation. ddPCR was employed to measure the intact proviral HIV DNA using the modified IPDA assay as described previously by Bruner et al.,^60^ providing estimates of intact provirus per one million CD4^+^ T cells. Additional details are discussed elsewhere.^34^

### 4.3 Genome‐wide DNA MC‐seq

Genome‐wide DNA methylation was conducted at the Yale Center for Genome Analysis according to the following steps.

#### 4.3.1 Methyl‐Seq target enrichment library prep

The concentration of genomic DNA was measured using fluorometry, while DNA quality was evaluated by determining the A260/A280 and A260/A230 ratios through spectrophotometry. DNA integrity and fragment size were confirmed utilizing a microfluidic chip analyzed on an Agilent Bioanalyzer. To prepare the whole genome sequencing libraries with indexed paired ends, the SureSelect XT Methyl‐Seq kit (Agilent; part#G9651B) was used according to the manufacturer's instructions. Specifically, DNA samples exceeding 350 ng were enriched for methylation sites using the custom SureSelect Methyl‐Seq Capture Library. The PCR amplification of these enriched and bisulphite‐converted libraries was performed with custom‐made indexed primers provided by IDT (Coralville, Iowa). The resulting dual‐indexed libraries were then quantified through quantitative PCR (qPCR) using the Library Quantification Kit from KAPA Biosystems (Part#KK4854). Insert size distribution was assessed with the Caliper LabChip GX system. Samples meeting a threshold concentration of 2 ng/µL proceeded to sequencing.

#### 4.3.2 Flow cell preparation and sequencing

Sample concentrations were adjusted to 10 nM and then loaded onto an Illumina NovaSeq flow cell, achieving a concentration sufficient for 40 million passing filter clusters per sample. Sequencing was performed using 100 bp paired‐end reads on an Illumina HiSeq NovaSeq, following Illumina's standard procedures. Data from the sequencing runs were transferred to the high‐performance computing cluster at the Yale Center for Genome Analysis. To ensure real‐time quality monitoring, a positive control prepared from the bacteriophage Phi X library (provided by Illumina) was spiked into each lane at a concentration of 0.3%. The 10 bp dual index was read during additional sequencing cycles that automatically proceeded after the first read.

#### 4.3.3 Preprocessing and QC

Signal intensities were converted to individual base calls during the sequencing run using the Real Time Analysis software. Sample de‐multiplexing and alignment to the human genome were conducted with Illumina's CASAVA 1.8.2 software suite. The sample error rate was maintained below 1%, and the distribution of reads per sample within a lane was required to be within reasonable tolerance. QC of sequencing reads was performed following standard procedures as previously described.^61^ The quality of sequence data was analysed using FastQC (ver. 0.11.8). Adapter sequences and low‐quality fragments at the 5′ and 3′ ends (phred score < 30) were removed using Trim_galore (ver. 0.6.3_dev).

Reads were aligned to the bisulphite‐converted human genome (hg19) using the Bismark pipelines (ver. v0.22.1_dev) with default parameters.^62^ On average, 87% of the sequences per sample were mapped to the genome. Quality‐trimmed paired‐end reads were transformed into either a bisulphite‐converted forward strand (C → T conversion) or a bisulphite‐treated reverse strand (G → A conversion of the forward strand). Only CpG sites with a coverage depth greater than 10 were considered for the final analysis to ensure the quality of CpG sites. This resulted in a total of 427 samples and 2 078 054 CpG sites available for epigenome‐wide association analysis.

Genes were annotated using the Homer annotatePeaks.pl script, including intergenic, 5′UTR, promoter, exon, intron, 3′UTR, transcription start site (TTS) and non‐coding regions. CpG island, shore, shelf and open sea annotations were defined by locally developed bash and R scripts based on the genomic coordinates (hg19) of CpG islands from the UCSC genome browser. CpG shores were defined as regions up to 2 kb from CpG islands, and CpG shelves as regions up to 2 kb from CpG shores.

### 4.4 Identification of CpG sites associated with HR

To ensure no other cell types were represented in the genomic DNA isolated from CD4^+^ T cells obtained from magnetic bead separation, we first estimated the PBMC cell type proportion using the Houseman method.^63^ The distribution of the HIV‐1 proviral DNA count (i.e., HR size) was normalised using a log10 transformation. Epigenome‐wide association analysis was performed using generalised linear models, in which methylation beta value at each CpG site was modelled as the dependent variable, and total HR size was modelled as the independent variable, while also adjusting for age, self‐reported race, cell type proportion and smoking status. We also adjusted the model for the lower detection limit of each HIV‐1 RNA viral load assay. The significance level was set at a FDR of 0.05. CpG sites that were evaluated for significant association with total HR were then re‐evaluated for association with intact HR_CD4_, 5′‐defective HR_CD4_ and 3′‐defective HR_CD4_ separately.

### 4.5 Identification of DNA methylation regions associated with total HR

Leveraging the bump‐hunting framework,^64^ we analysed DNA methylation regions (as opposed to individual CpG sites) that were associated with total HR_CD4_. Bump hunting provides the advantage of effectively modelling measurement errors, removing batch effects and detecting regions of interest for continuous measures such as HR_CD4_. We modified the bump hunting pipeline of Jaffe et al.^64^ to increase the sensitivity for detecting methylation regions. In the regression model, instead of using methylation β value at each CpG site, we used the *t*‐statistic value that was derived from EWAS regressed on the total HR_CD4_. The *p* value of each region was then estimated following the method proposed by Lui et al.^65^ A significant region was defined as having at least two consecutive CpG sites and an adjusted *p* < .05.

### 4.6 Gene set enrichment analysis

Genes adjacent to CpG sites were selected for gene set enrichment analysis. A cutoff of *p* < .05 was used to ensure enough genes were selected for enrichment analysis. We used the Hallmark database from the Molecular Signatures Database (https://www.gsea‐msigdb.org/gsea/msigdb/) and the GO database for the enrichment analyses.

## AUTHOR CONTRIBUTIONS

K. X. oversaw the study, including DNA methylation sequencing data collection, data processing, statistical analyses and manuscript preparation. X. Z. conducted data processing and analysis and contributed to manuscript preparation. B. E. A. and K. A. handled CD4^+^ T cell isolation, genomic DNA isolation and HIV‐1 reservoir detection. B. C. Q., G. P. P. and D. B. H. participated in analytic strategy and manuscript preparation. D. K. P., H. H. B., C. D. L., E. T. G., M. H. C., N. M. A., M. H. K. and P. C. T. participated in the study's conduct, including protocol development, participant recruitment, follow‐up and biospecimen collection. A. V. contributed to manuscript preparation. V. C. M. contributed to the interpretation of the findings and preparation of the manuscript. E. O. J. and B. E. A. contributed to the study design, interpretation of findings and manuscript preparation. All authors had full access to all the data in the study, had read and approved the final manuscript and had accepted responsibility to submit it for publication.

## CONFLICTS OF INTEREST STATEMENT

V. C. M. has received investigator‐initiated research grants (to the institution) and consultation fees (both unrelated to the current work) from Eli Lilly, Bayer, Gilead Sciences and ViiV. The remaining authors declare that they have no competing interests.

## Supporting information



Supporting information

Supporting information

Supporting information

## Data Availability

Summary statistics of the epigenome‐wide analysis are presented in the Supporting Information tables. Access to individual‐level DNA methylation data from the MACS/WIHS Combined Cohort Study Data (MWCCS) may be obtained upon review and approval of a MWCCS concept sheet. Links and instructions for online concept sheet submission are on the study website (https://statepi.jhsph.edu/mwccs/).
