# Supplementary figures and images for: Aberrant DNA methylation of genes regulating CD4+ T cell HIV‐1 reservoir in women with HIV
Source: Clin Transl Med. 2025 Mar 11;15(3):e70267. doi: 10.1002/ctm2.70267 (PMC11896887; doi:10.1002/ctm2.70267)

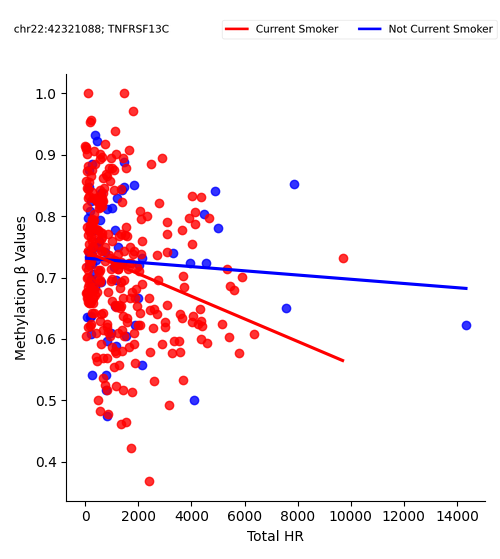

Supplement: Supplementary file 2 — Supporting information [file CTM2-15-e70267-s003.png]

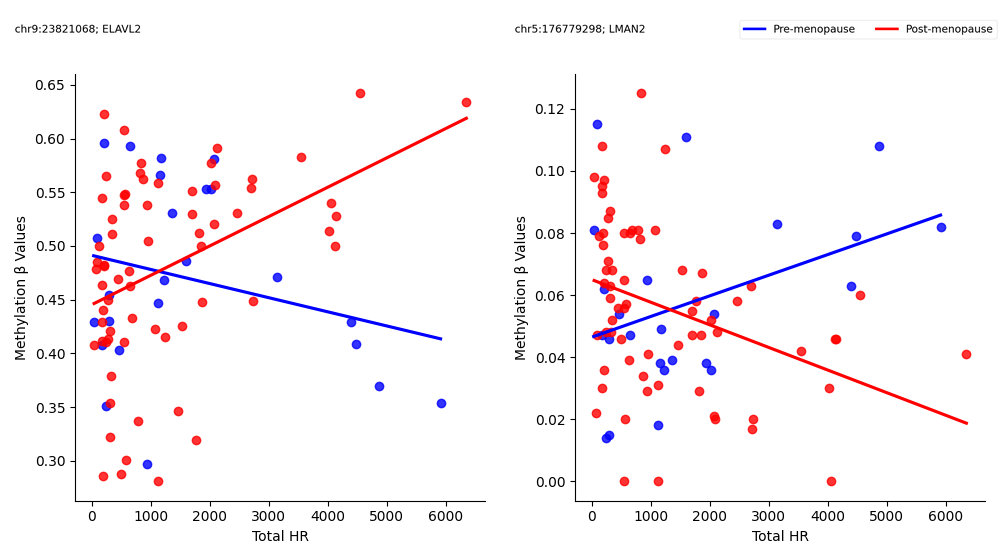

Supplement: Supplementary file 3 — Supporting information [file CTM2-15-e70267-s001.png]
